# Effect of esketamine on the ED_50_ of propofol for successful insertion of ureteroscope in elderly male patients: a randomized controlled trial

**DOI:** 10.1186/s12871-024-02580-z

**Published:** 2024-05-31

**Authors:** Xin Luo, Wen-Wen Hao, Xue Zhang, Yu-Xuan Qi, Li-Xin An

**Affiliations:** 1 Department of Anesthesiology, Beijing Friendship Hospital, Capital Medical University, No.95 Yongan Road, Xicheng District, Beijing, 100050 China; 2 Department of Anesthesiology, Jingmei Group General Hospital, Beijing, China

**Keywords:** Esketamine, ED_50_, Propofol, Ureteroscope, Elderly patients

## Abstract

**Background:**

Propofol is effective and used as a kind of routine anesthetics in procedure sedative anesthesia (PSA) for ureteroscopy. However, respiratory depression and unconscious physical activity always occur during propofol-based PSA, especially in elderly patients. Esketamine has sedative and analgesic effects but without risk of cardiorespiratory depression. The purpose of this study is to investigate whether esketamine can reduce the propofol median effective dose (ED_50_) for successful ureteroscope insertion in elderly male patients.

**Materials and methods:**

49 elderly male patients undergoing elective rigid ureteroscopy were randomly divided into two groups: SK Group (0.25 mg/kg esketamine+propofol) and SF Group (0.1 µg/kg sufentanil+propofol). Patients in both two groups received propofol with initial bolus dose of 1.5 mg/kg after sufentanil or esketamine was administered intravenously. The effective dose of propofol was assessed by a modified Dixon’s up-and-down method and then was adjusted with 0.1 mg/kg according to the previous patient response. Patients’ response to ureteroscope insertion was classified as “movement” or “no movement”. The primary outcome was the ED_50_ of propofol for successful ureteroscope insertion with esketamine or sufentanil. The secondary outcomes were the induction time, adverse events such as hemodynamic changes, hypoxemia and body movement were also measured.

**Result:**

49 patients were enrolled and completed this study. The ED_50_ of propofol for successful ureteroscope insertion in SK Group was 1.356 ± 0.11 mg/kg, which was decreased compared with that in SF Group, 1.442 ± 0.08 mg/kg (*P* = 0.003). The induction time in SK Group was significantly shorter than in SF Group (*P* = 0.001). In SK Group, more stable hemodynamic variables were observed than in SF Group. The incidence of AEs between the two groups was not significantly different.

**Conclusion:**

The ED_50_ of propofol with esketamine administration for ureteroscope insertion in elderly male patients is 1.356 ± 0.11 mg/kg, significantly decreased in comparsion with sufentanil.

**Trial registration:**

Chinese Clinical Trial Registry, No: ChiCTR2300077170. Registered on 1 November 2023. Prospective registration. http://www.chictr.org.cn.

## Introduction

Most urological surgeries are performed using minimally invasive techniques or cystoscopy in narrow and limited spaces, and most patients undergoing urological surgery are elderly people who also suffer from other diseases [1]. Therefore, in addition to providing sufficient anesthesia, anesthesiologists should also consider various factors such as age, comorbidities, functional status, surgical duration, expected blood loss, and surgical scope to optimize surgical outcomes [2].

For some short and less stimulating procedure such as transurethral ureteroscope lithotripsy (URL) or cystoscopy, anesthesiologists are often willing to choose procedure sedative anesthesia (PSA). On the one hand, it avoids the inhibition of circulation and long wake-up time in elderly patients by general anesthesia drugs, on the other hand, it also avoids the long spinal anesthesia operation time caused by elderly osteoporosis [3, 4]. Propofol has been widely used for induction and maintenance of intravenous anesthesia in PSA with unique advantages such as short onset time, with a satisfactory and rapid postoperative recovery [2, 5]. However, elderly patients with medical comorbidities also have a certain risk of developing hypotension, bradycardia, hypoxemia, movement and reduced surgical satisfaction during PSA [2, 6]. Adjuvant drugs were usually needed to enhance the anesthetic efficacy and reduce the incidence of side effects from a single dose or continuous infusion of propofol [7].

Several adjuvant drugs (such as opioids, dexmedetomidine, ketamine, etc.) have been preferred in combination to reduce the propofol requirement and minimize associated adverse events in sedation procedure [8]. Opioids and benzodiazepine are the most common drugs in addition to propofol during PSA with side effects including dose-dependent hypotension and hypoxemia [9, 10]. Dexmedetomidine exerts an anesthetic sparing effect and has less influence on respiratory system but may result in hemodynamic instability and prolong sedation [11]. Ketamine has anti-hyperalgesic effects and an opioid-sparing effect to decrease pain intensity, even though schizophrenia-like symptoms often occur [12]. Therefore, ideal adjuvant drugs with minimal side effects and reduced adverse reactions to propofol administration in elderly patients is needed.

Esketamine is a novel noncompetitive N-methyl-D-aspartate receptor antagonist with sedative, analgesic and sympathomimetic effects but does not induce respiratory or circulatory depression [13, 14]. The available evidence indicates that esketamine can reduce propofol requirement, provide improved anesthetic effect with reduced adverse events for gastroendoscopy and fibronchoscopy in elderly patients [7, 15]. However, few studies have been reported the influence of intravenous esketamine administration on the propofol requirement for ureteroscope insertion in elderly male patients. The goal of our investigation is to estimate the ED_50_ of propofol combined with intravenous esketamine for success ureteroscope insertion in elderly patients and find out whether the addition of intravenous esketamine can reduce the ED_50_ of propofol.

## Methods

### Ethics statement

This was a prospective, double-blinded randomized controlled study conducted in Beijing Friendship Hospital of Capital Medical University. This study protocol was approved by the Institutional Ethics Committee of Beijing Friendship Hospital, Capital Medical University (Approval No: 2023-P2-246-02), registered on the Chinese Clinical Trial Registry (http://www.chictr.org.cn; registration number: ChiCTR2300077170, 1 November 2023).

### Inclusion and exclusion criteria

Elderly male patients scheduled for painless ureteroscopy were enrolled and signed the informed consent if they accorded with the inclusion criteria: (1) body mass index (BMI) 18 ∼ 28 kg/cm^2^; (2) ≥ 60 years old; (3) gender: male; (4) American Society Anesthesiologists physical status (ASA) I ∼ III. The exclusion criteria included: (1) allergic to the trial medication; (2) psychiatric disorders; (3) severe liver or kidney disorders; (4) uncontrolled hypertension or malignant hypertension; (5) severe diseases of circulatory system; (6) acute respiratory tract infections or other chronic respiratory disorders; (7) high intracranial pressure or high intraocular pressure. Discharge criteria: (1) change of surgery type when entering operation room; (2) accidents such as severe hemodynamic instability or high cough sensitivity after injecting intravenous anesthetics; (3) inability to tolerate ureteroscopy under intravenous anesthesia; (4) other unexpected circumstances. If patient refused to continue the trial or violated the scheme, they were also excluded from the trial.

### Randomization and masking

50 patients were randomly divided into two groups: SK group (0.25 mg/kg esketamine, *n* = 25) and SF Group (0.1 µg/kg sufentanil, *n* = 25). The patients, anesthesiologists and urologists were all blinded to the grouping. A random sequence of numbers was generated by a computer and divided into two groups at a ratio of 1:1 into sealed envelopes. After the patients were included, the groups were randomly allocated and the envelope were opened. Then an anesthesiologist nurse would prepare the experimental drugs in colorless syringe, seal and give them to the anesthesiologist according to the numbers placed in the the envelopes.

### Standard anesthesia procedures

The patients fasted from food and water for 8 h before ureteroscopic procedures according to our routine practice and non-invasive blood pressure (NBP), pulse oxygen saturation (SpO_2_), heart rate (HR) and electrocardiography (ECG) were continuously monitored after entering operating room. Patients were placed in the lithotomy position and an intravenous access was established. Before given intravenous anesthetics, patients inhaled 6 L/min oxygen continuously via a facemask and received scopolamine 0.3 mg via a vein.

The experimental drugs, including both esketamine and sufentanil solutions, were diluted into 10 ml and prepared by a specialized nurse. The patients received 0.25 mg/kg esketamine in SK group and 0.1 µg/kg sufentanil in SF group, respectively. 3 min after experimental drugs administration, an initial bolus of propofol (1.5 mg/kg in both groups according to our previous experience) was intravenously injected in 30–60 s for anesthesia induction.

The modified observer’s assessment of alertness/sedation (MOAA/S) score [16] (5: response readily to name spoken; 4: lethargic response; 3: response after name called loudly; 2: response after mild to moderate shaking; and 1: response to trapezius squeeze) was utilized to evaluate the sedation depths of patients. After propofol was administered intravenously and the patients’ MOAA/S score ≤ 1, the ureteroscope insertion was performed by an experienced urologist. The patient’s responses to the ureteroscope insertion were classified as either ‘movement’ or ‘no-movement’. The ‘movement’ response was defined as movement of limbs to the whole body requiring a propofol increment. The conditions of endoscope insertion were only evaluated at the first attempt and a bolus of 20 mg propofol, as a rescue medication, was administered immediately once movement responses or condition of MOAA/S score ≥ 2 occurred during the procedure.

All adverse events during propofol sedation and ureteroscopy were recorded. Hypotension was determined when mean artery pressure (MAP) decreased below 65 mmHg, and 5 mg ephedrine was intravenously administered immediately if necessary. Bradycardia was determined when HR decreased below 45 beats/min, and 0.25–0.5 mg atropine was intravenously injected as needed. Hypoxemia was determined if SpO_2_ decreased to lower than 90%, and maybe needed assisted ventilation with a facemask even an artificial ventilation support.

### Outcome endpoints

The primary endpoint was the ED_50_ of propofol for successful ureteroscope insertion in elderly male patients. ED_50_ of propofol was evaluated according to the modified Dixon’s up-and-down method (MDUDM) [17]. The previous studies had determined ED_50_ of propofol in combination with lidocaine for gastrointestinal endoscopy sedation in adult patients [18] and ED_50_ of propofol combined with single dose of ketamine during the UGI endoscopy in elderly patients [19]. As mentioned above, 1.5 mg/kg propofol was set for the first patient in our study. According to MDUDM, if the response of the first participant was ‘no-movement’, the induction dose of propofol would be decreased by 0.1 mg/kg in the subsequent patient, otherwise increased. A crossover point was determined when patient’s response was changed from ‘movement’ to ‘no-movement’. After seven crossover points were obtained, patient recruitment was stopped.

The secondary endpoints were the induction time and all adverse events from propofol sedation until the end of ureteroscopy procedure.

### Statistical analysis

Previous studies have reported that anesthesia achievement using the up-and-down methodology routinely needs 20–40 patients each group [15, 20]. Given a dropout rate of 10%, the estimated sample size was set as 25 patients per group and a total of 50 patients will be enrolled.

Statistical analysis of data was performed using SPSS statistical software (version 26.0, SPSS Inc, Chicago, IL, USA) and *P* values < 0.05 indicated a significant difference. For continuous variables, the normality test was performed by using the Kolmogorov–Smirnov test firstly. Data with a normal distribution was expressed as mean ± SD and intergroup comparisons were analyzed with an independent Student’s t-test. For data with a non-normal distribution, medians (inter quartile range, IQR) was used for expression and intergroup comparisons were performed by Wilcoxon signed-rank test. Categorical variables were expressed as number and/or percentage and the Fisher’s exact test or χ^2^ test was used to analyze the data according to the frequency.

## Results

**Fig. 1:**
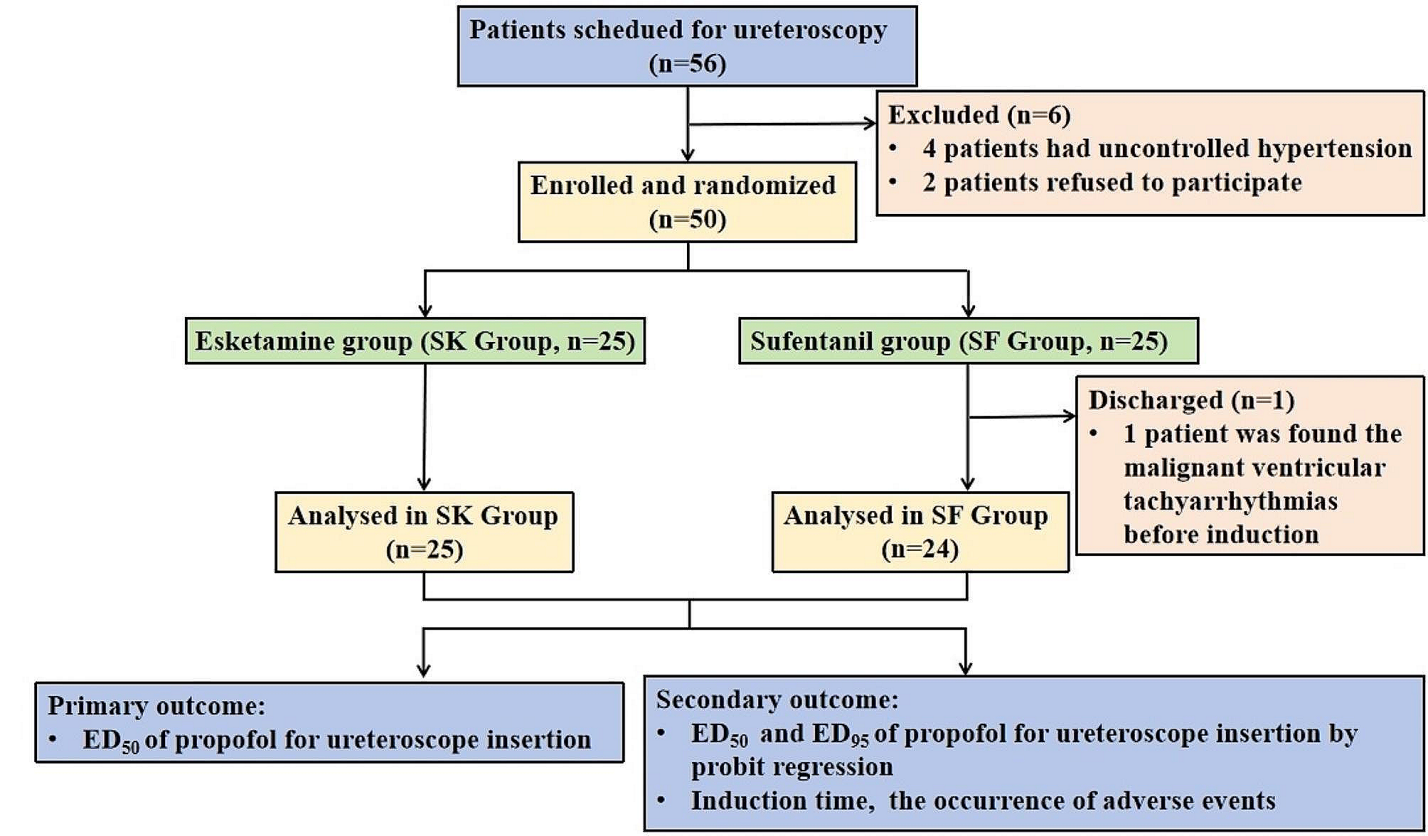
The flow chart of included and excluded patients

A total of 49 patients were enrolled and completed the study from November 10, 2023 to February 10, 2024. Eventually, 25 patients in SK Group and 24 patients in SF Group were all shown in the flow chart in Fig. 1 and there was no significant difference in demographic data between groups (*P* > 0.05) in Table 1.

**Table 1:** Demographic data of patients

Parameters	SK Group (*n* = 25)	SF Group (*n* = 24)	*P* values
Age (years)	69.3 ± 5.9	68.8 ± 5.3	0.764
BMI (kg/cm^2^)	24.9 ± 1.7	23.9 ± 2.0	0.053
ASA (I/II/III)	2/18/5	1/17/6	1.000
Smoking (Y/N)	3 (12.0%)	2 (8.3%)	1.000
Alcohol use (Y/N)	5 (20.0%)	3 (12.5%)	0.702
Comorbidities			
Hypertension	12 (48.0%)	12 (50.0%)	1.000
Diabetes	9 (36.0%)	4 (16.7%)	0.125
Coronary heart disease	6 (24.0%)	1 (4.2%)	0.098
Chronic obstructive pulmonary disease	0 (0.0%)	1 (4.2%)	0.490
Lacunar infarction	0 (0.0%)	2 (8.3%)	0.235

Data are presented as the mean ± SD or number of patients (%)

Notes: SK Group: esketamine group; SF Group: sufentanil group

**Fig. 2:**
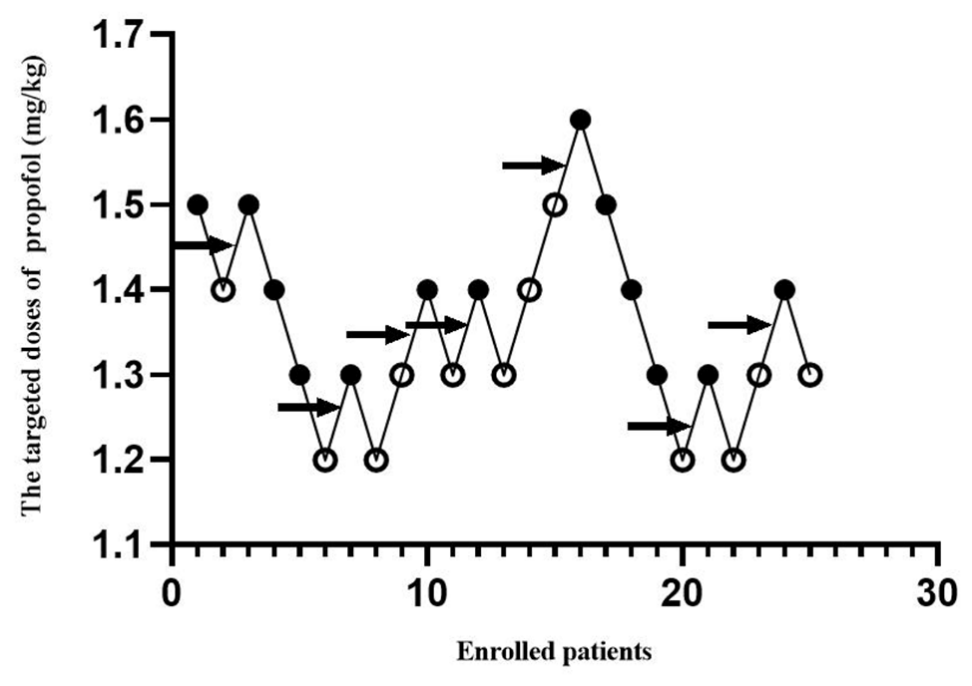
Responses of patients with the modified Dixon’s up-and-down method. Responses of 25 enrolled patients to ureteroscope insertion in SK Group and their initial doses of propofol are shown. Arrow indicates the midpoint dose of all independent pairs of patients who manifested crossover from ‘movement’ (○) to ‘no movement’ (●) responses

**Fig. 3:**
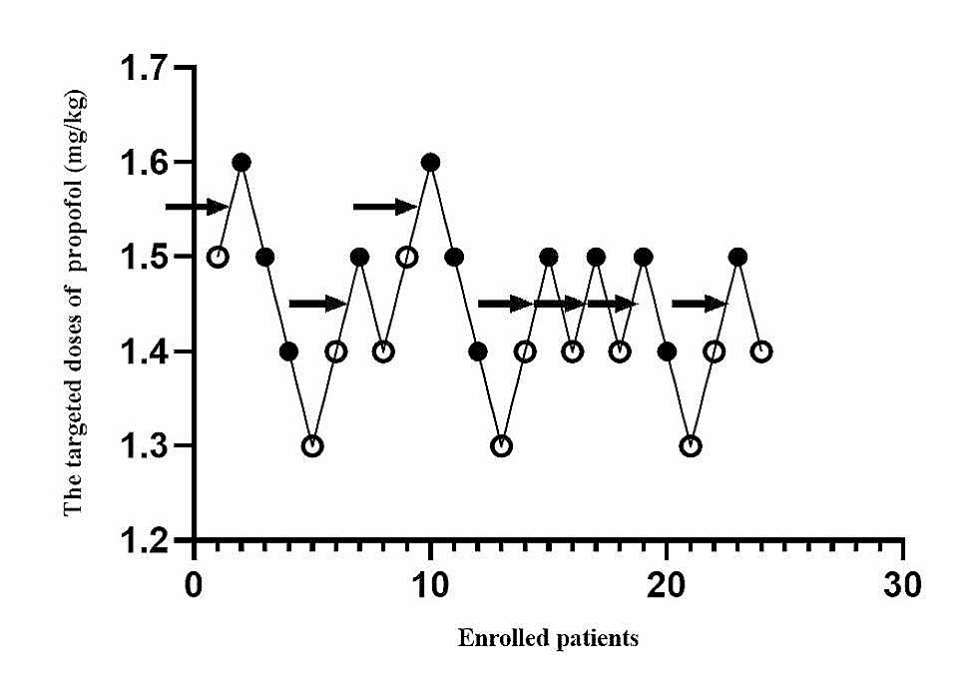
Responses of 24 enrolled patients to ureteroscope insertion in SF Group and their initial doses of propofol are shown. Arrow indicates the midpoint dose of all independent pairs of patients who manifested crossover from ‘movement’ (○) to ‘no movement’ (●) responses

**Fig. 4:**
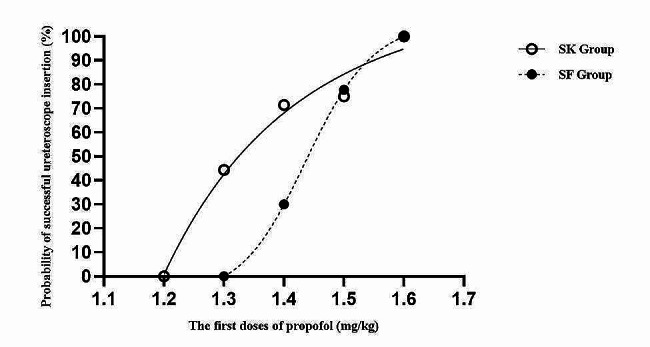
Dose-response curves of propofol for successful ureterooscope insertion

The ED_50_ of propofol for successful ureteroscope insertion in SK Group was 1.356 ± 0.11 mg/kg, which was decreased compared with that in SF Group, 1.442 ± 0.08 mg/kg (Figs. 2 and 3). The dose-response curves of propofol for successful ureteroscope insertion calculated and drawn from the probit regression analysis were shown in Fig. 4. ED_50_ and ED_95_ of propofol for successful ureteroscope insertion in SK Group were 1.347 mg/kg (95% confidence interval, 1.213–1.461 mg/kg) and 1.566 mg/kg (95% confidence interval, 1.455–2.497 mg/kg), respectively. By the same method as described above, ED_50_ and ED_95_ of propofol for successful ureteroscope insertion in SF Group were 1.442 mg/kg (95% confidence interval, 1.379–1.503 mg/kg) and 1.557 mg/kg (95% confidence interval, 1.498–1.883 mg/kg), respectively.

**Fig. 5:**
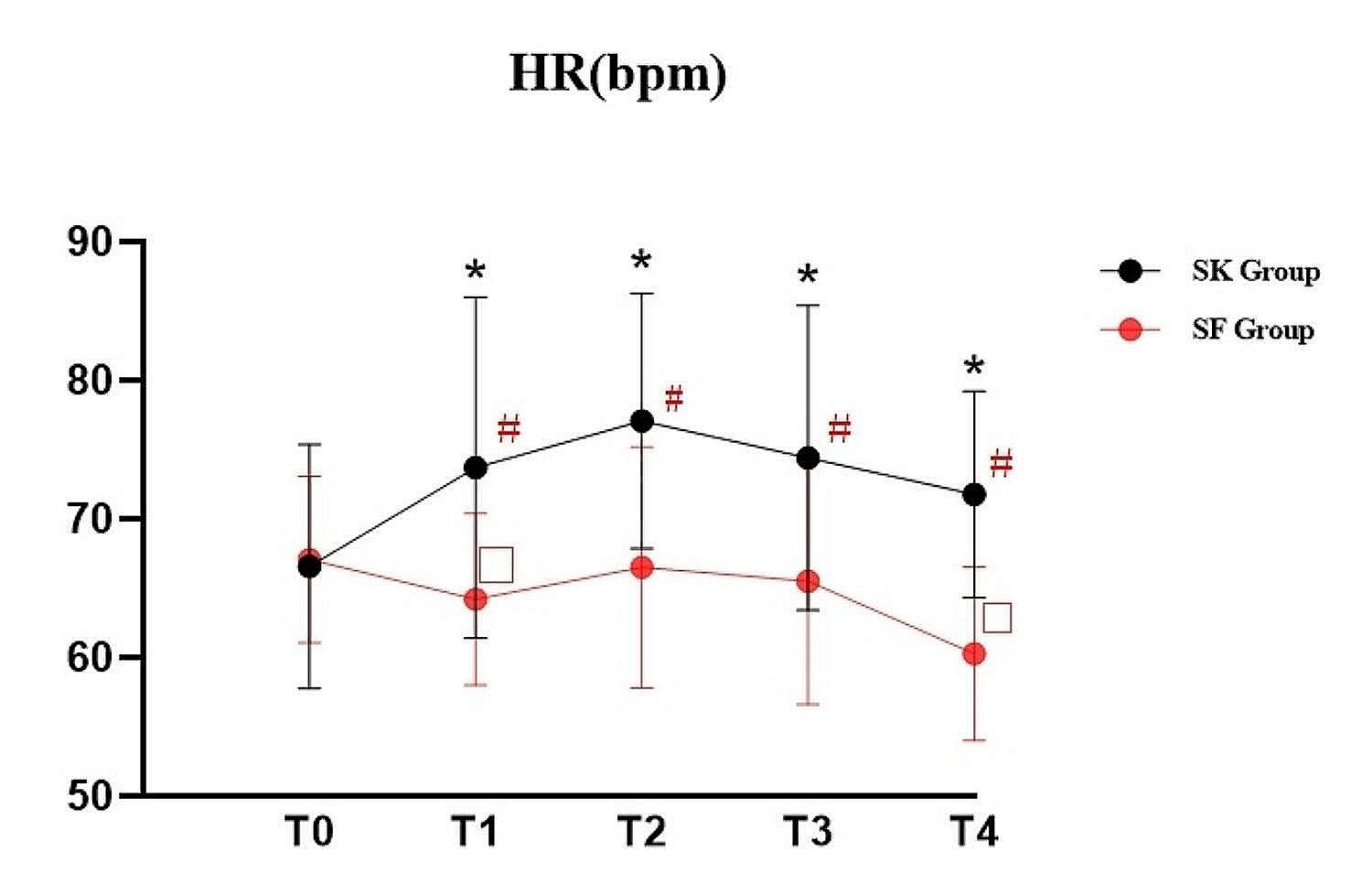
Heart rate (HR) change: T0: before induction; T1: after experimental drugs administration; T2: after propofol injection; T3: ureteroscope insertion; T4: at the end of procedure; *P < 0.05, SK group vs. SF group;#*P* < 0.01, the values of different time points vs. T0 in SK group; ♦*P* < 0.01, the values of different time points vs. T0 in SF group

**Fig. 6:**
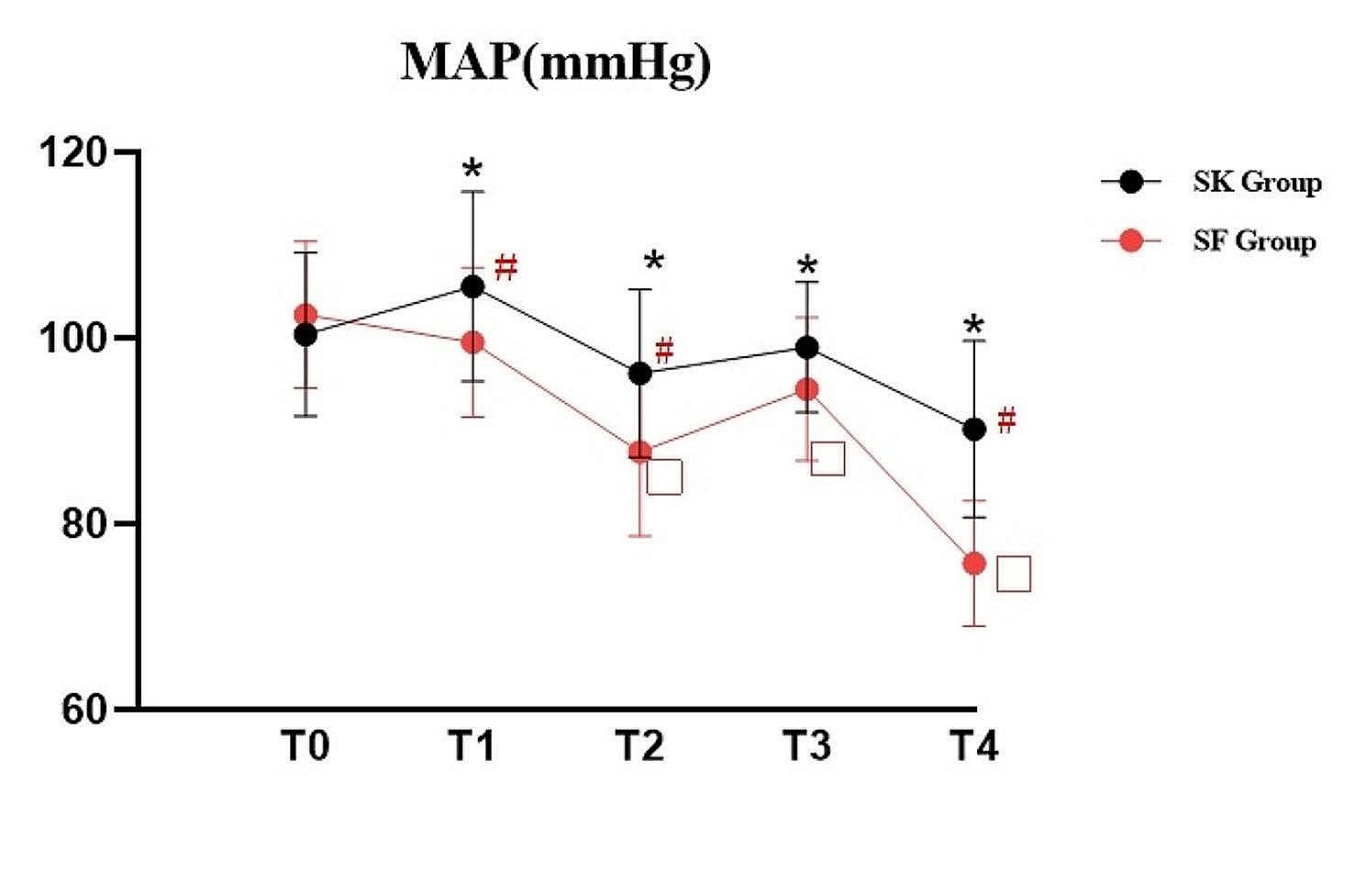
Mean arteral pressure (MAP) change: T0: before induction; T1: after experimental drugs administration; T2: after propofol injection; T3: ureteroscope insertion; T4: at the end of procedure; *P < 0.05, SK group vs. SF group;#*P* < 0.01, the values of different time points vs. T0 in SK group; ♦ *P* < 0.01, the values of different time points vs. T0 in SF group

The HR and MAP changes at different time points were shown in Figs. 5 and 6. HR after experimental drugs administration, after propofol injection, at ureteroscope insertion and at the end of procedure were significantly higher in SK Group compared to SF Group (*P <* 0.05). In the meantime, HR after experimental drugs administration, after propofol injection, at ureteroscope insertion and at the end of procedure were significantly higher than the baseline value before induction in SK Group (*P <* 0.01). In SK Group, significant elevation of HR were found in other time points and the MAP value after experimental drugs administration was significantly higher compared with the value before induction; significant decline of MAP was found after propofol injection and at the end of procedure. In SF Group, significant decline of HR were found after experimental drugs administration and at the end of procedure; meanwhile, similar reduction of MAP were found after propofol injection, at ureteroscope insertion and at the end of procedure (*P <* 0.05).

The induction time in SK Group was significantly shorter than in SF Group (*P* = 0.001). The number of hypoxemia between the two groups was not statistically significant difference. However, in comparison with SF Group, the number of patient’s SpO_2_ ≤ 95% was notably lower in SK Group (*P <* 0.05). No significant differences were observed in body movement between the two groups (*P*>0.05) and no other adverse events occurred during propofol sedation or after procedure (Table 2).

**Table 2:** Induction time, occurrence of AEs during sedation and ureteroscope insertion

Parameters	SK Group (*n* = 25)	SF Group (*n* = 24)	*P* values
Induction time (s)	22.0 (17.5, 25.5)	34.5 (22.8, 43.0)	**0.001**
Propofol sedation			
SpO_2_ > 95%	17	8	**0.047**
90%< SpO_2_ ≤ 95%	7	12	
SpO_2_ ≤ 90%	1	4	
Ureteroscope insertion			
No-movement	14	12	0.674
Movement	11	12	

Data are presented as the median (IQR) or the number of patients (%)

Notes: SK Group: esketamine group; SF Group: sufentanil group

## Discussion

It is increasingly common for elderly patients to undergo ureteroscopy for various reasons, and the effectiveness of esketamine combined with propofol in elderly patients undergoing ureteroscopy is still unknown. In our study, the ED_50_ of propofol combined with esketamine for successful endoscope insertion in adult patients, was significantly reduced compared with sufentanil. The induction time was shorter and incidence of SpO_2_ ≤ 95% was lower in esketamine group than that in sufentanil group.

This was the first to determine the effective dose of propofol for successful ureteroscope insertion and evaluate the influence of intravenous esketamine compared with sufentanil on the ED_50_ of propofol in elderly male patients. In our study, the main endpoint, ED_50_ of propofol combined with 0.25 mg/kg esketamine for successful ureteroscope insertion was 1.356 ± 0.11 mg/kg and combined with 0.1 µg/kg sufentanil was 1.442 ± 0.08 mg/kg. This outcome demonstrated that co-administration with esketamine could effectively decrease propofol requirement than co-administration with sufentanil. At first, the classic MDUDM was the only method used to determine the dose of propofol required for 50% success in ureteroscopy among elderly male patients. However, there is still a failure of another 50% patients for ureteroscope insertion, then the probit regression analysis was utilized to determine the ED_95_ of propofol combined with esketamine or sufentanil (1.566 mg/kg vs. 1.557 mg/kg), which can inhibit most ‘movement’ responses to ureteroscope insertion in elderly male patients.

The secondary endpoints of this study were the induction time and perioperative hemodynamic changes. The results showed that propofol administration with esketamine could provide satisfactory sedative effect and more stable perioperative hemodynamic variables compared with co-administration with sufentanil, which was consistent with previous studies [21, 22]. Zheng et al. found that addition of 0.25 mg/kg esketamine can shorter induction time and improve safety by reducing the dosage of anesthetics [22]. The esketamine’s advantages of reduction of adverse reactions may also be related to lower dosage of propofol injection.

Esketamine has stronger sedative and analgesic properties correlated with its effect on the NMDA-receptor. Hemodynamic and respiratory events are less frequent than other sedatives and analgesics, due to an increase induced by esketamine in sympathetic tone [23]. Recent studies demonstrated that 0.15 ∼ 0.5 mg/kg esketamine for elderly patients could not only decrease propofol requirement, but also lower the risk of delayed postoperative recovery due to over-dose propofol medication [7, 11, 21]. Another clinical study demonstrated that the mean induction dosage of propofol for elderly patients was 1.7 mg/kg, which was consistent with our results [24]. In the study of Zheng et al’s [19], it’s reported that the ED_50_ and ED_95_ of propofol with a single dose of 0.3 mg/kg esketamine were 1.479 mg/kg (95% CI: 1.331 ∼ 1.592 mg/kg) and 1.738 mg/kg (95% CI: 1.614 ∼ 2.487 mg/kg), respectively, significantly lower than those in our study. The different findings may be attributed to the lower dose of esketamine administered and stronger stimulation of the procedure performed in our study. Additionally, 18 males and 14 females were enrolled in Zheng et al.‘s study differed from our study just enrolling males. The above distinctions of population may also cause two studies’ variation. In Yang et al’s [7] study, both 0.25 mg/kg and 0.5 mg/kg esketamine in combination with propofol could provide an adequate level of sedation and analgesia with less propofol consumption. Moreover, computer-controlled target-controlled infusion (TCI) was applied to determine ED_50_ of propofol with disadvantages of longer time consumption to achieve the target concentration and indirect injection compared with the up-down method. With potentially psychotomimetic effects correlated with a relatively large dose of esketamine, intravenous 0.25 mg/kg was evaluated as a safe dosage for the elderly population and an up-down method was applied in our study to explore the ED_50_.

Hypoxemia and hypotension are the frequently reported cardiorespiratory complications induced by dose-dependent propofol administration for the elderly [25]. Previous studies have demonstrated that combination of low-dose esketamine with propofol can effectively reduce the incidence of respiratory depression and maintain hemodynamic stability by reducing propofol consumption [19, 26]. In our study, the elevation of HR and MAP in SK Group after experimental drugs administration compared to SF group may contributed to the sympathomimetic effect of esketamine [14, 15, 23]. In SK Group, HR values increased not only after experimental drugs, but also in other time points which were also partly attibuted to the susceptible cardiovascular sympathetic response to esketamine. MAP values within the groups declined after esketamine or sufentanil combined with propofol injection. Lower-dose esketamine exert an adequate transient elevation effect on blood presure, but the influence still cannot absolutely counteract MAP decline produced by propofol administration. Even though there was no statistical difference between the occurrence of the number of patients’ SpO2 ≤ 90% and movement in both groups, the number of patients’ SpO2 ≥ 90% was significantly lower in the SF Group. It seems that addition dose of esketamine could relieve the degree of respiratory depression with reduction of the bolus propofol consumption. There was no hypotension occured and hemodynamic status was more stable in SK Group. In addition to lower requirement of propofol sedation, esketamine has unique sympathomimetic properties which may partially counter propofol-induced haemodynamic depression. The outcomes were adhered to our original hypothesis.

There are two limitations in our study. Given type and duration of ureteroscopy identified as independent risk factors for procedure-related complications, our results should not be applied in other types of therapeutic procedures. Then, MOAA/S score was the only evaluation indicator applied to measure sedation level, which may not access depth of anesthesia as real. Thus, BIS or EEG monitoring, continuous objective indicators, are considered for application further.

## Conclusions

In conclusion, the ED_50_ of propofol with esketamine administration for ureteroscope insertion in elderly male patients significantly decreased compared with sufentanil. At the same time, intravenous esketamine can effectively reduce propofol requirement, shorten induction time and provide more hemodynamic stability with less adverse cardiovascular events.

## Data Availability

The data sets used and/or analyzed during the present study are available from the corresponding author on reasonable request. The email of corresponding author is anlixin8120@163.com.

## Abbreviations

URL: Ureteroscope lithotripsy
PSA: Procedure sedative anesthesia
BMI: Body Mass Index
ASA: American Society Anesthesiologists
NBP: Non-invasive blood pressure,
HR: Heart rate
SpO_2_: Pulse oximetry
ECG: Electrocardiography
MOAA/S: Modified observer’s assessment of alertness/sedation
MAP: Mean artery pressure
MDUDM: Modified Dixon’s up-and-down method
UGI: Upper gastrointestinal
ED_50_: 50% effective dose
ED_95_: 95% effective dose
NMDA receptor: N-methyl-D-aspartate receptor
TCI: Computer-controlled target-controlled infusion
BIS: Bispectral index
EEG: Electroencephalographic
